# Updated US Prevalence Estimates for Chronic Kidney Disease Stage and Complications Using the New Race-Free Equation to Estimate Glomerular Filtration Rate

**DOI:** 10.1001/jamanetworkopen.2022.0460

**Published:** 2022-02-15

**Authors:** Carl P. Walther, Wolfgang C. Winkelmayer, Sankar D. Navaneethan

**Affiliations:** 1 Selzman Institute for Kidney Health, Section of Nephrology, Department of Medicine, Baylor College of Medicine, Houston, Texas; 2 Section of Nephrology, Michael E. DeBakey Veterans Affairs Medical Center, Houston, Texas; 3 Institute of Clinical and Translational Research, Baylor College of Medicine, Houston, Texas

## Abstract

This cross-sectional study investigates US population changes in chronic kidney disease (CKD) G stage and 4 CKD-related complications, comparing the 2021 equation for estimating glomerular filtration rate with the 2009 equation, which included a race coefficient.

## Introduction

Estimating glomerular filtration rate (eGFR) from serum creatinine is common in medicine. The 2009 Chronic Kidney Disease–Epidemiology Collaboration (CKD-EPI) equation uses age, sex, race (Black vs non-Black), and creatinine level. Concerns were raised that the use of race in estimating GFR could contribute to care inequities.^1^ CKD-EPI recently derived an equation for eGFR from serum creatinine level without a race coefficient, the adoption of which is recommended by a national task force.^2,3^ Validity of CKD staging using eGFR thresholds (ie, CKD G stages) is in part supported by CKD-related complications at more severe stages.^4^ Changes in G stage with the new equation could affect diagnosis and treatment for many persons. We investigated US population changes in CKD G stage and in 4 CKD-related complications, comparing the race-free serum creatinine–based 2021 CKD-EPI equation with the 2009 equation.

## Methods

In this cross-sectional study, we used data from the US National Health and Nutrition Examination Survey (NHANES 2011-2018). NHANES was approved by the National Center for Health Statistics research ethics review board, and participants gave written informed consent. This analysis used publicly available deidentified data and was not considered human participant research per Baylor College of Medicine institutional review board policy. Appropriate survey methods were used.^5^ This cross-sectional study followed the Strengthening the Reporting of Observational Studies in Epidemiology (STROBE) reporting guideline.

We included NHANES participants aged 20 years and older and classified them by self-reported race into Black vs non-Black groups (Mexican American, other Hispanic, non-Hispanic Asian, non-Hispanic White, and other race, including multiracial, ie, the categories used in the 2009 equation). We calculated eGFR using serum creatinine–based CKD-EPI equations (2009 and 2021); G stage^6^ was calculated for eGFRs of less than 60 mL/min/1.73 m^2^. Anemia was defined as hemoglobin level of less than 12 g/dL in female participants and less than 13 g/dL in male participants (to convert hemoglobin to grams per liter, multiply by 10.0), acidosis as serum bicarbonate levels of 22 mEq/L or lower (to convert to millimoles per liter, multiply by 1), hyperphosphatemia as serum phosphate levels of 4.5 mg/dL or greater, and hypertension as systolic blood pressure of 140 mm Hg or greater or diastolic blood pressure of 90 mm Hg or greater. Multiple imputation was performed for missing laboratory values (creatinine was missing in 10.3% of participants). Analyses were performed with Stata version 14.2 (StataCorp). Additional details are in the eAppendix in the Supplement.

## Results

Overall, 39 156 individuals participated in NHANES 2011-2018. An estimated 18.1 (95% CI, 16.6-19.6) million adults had an eGFR of less than 60 ml/min/1.73 m^2^ using either equation. The mean (SD) age was 71 (13) years, and an estimated 10.5 (95% CI, 9.5-11.6) million were female. An estimated 5.5 (95% CI, 4.9-6.2) million adults were reclassified in G stage with the 2021 equation: 1.0 (95% CI, 0.8-1.2) million into a more severe stage and 4.5 (95% CI, 3.8-5.2) million into a less severe stage. All persons reclassified into more severe stages self-reported as Black, and all persons reclassified into less severe stages self-reported as non-Black. Of all Black adults, 3.9% (95% CI, 3.3%-4.4%) were recategorized into a more severe stage (Figure). Of all non-Black adults, 2.2% (95% CI, 1.9%-2.5%) were recategorized into a less severe stage. CKD-related complications are shown in the Table.

**Figure.**
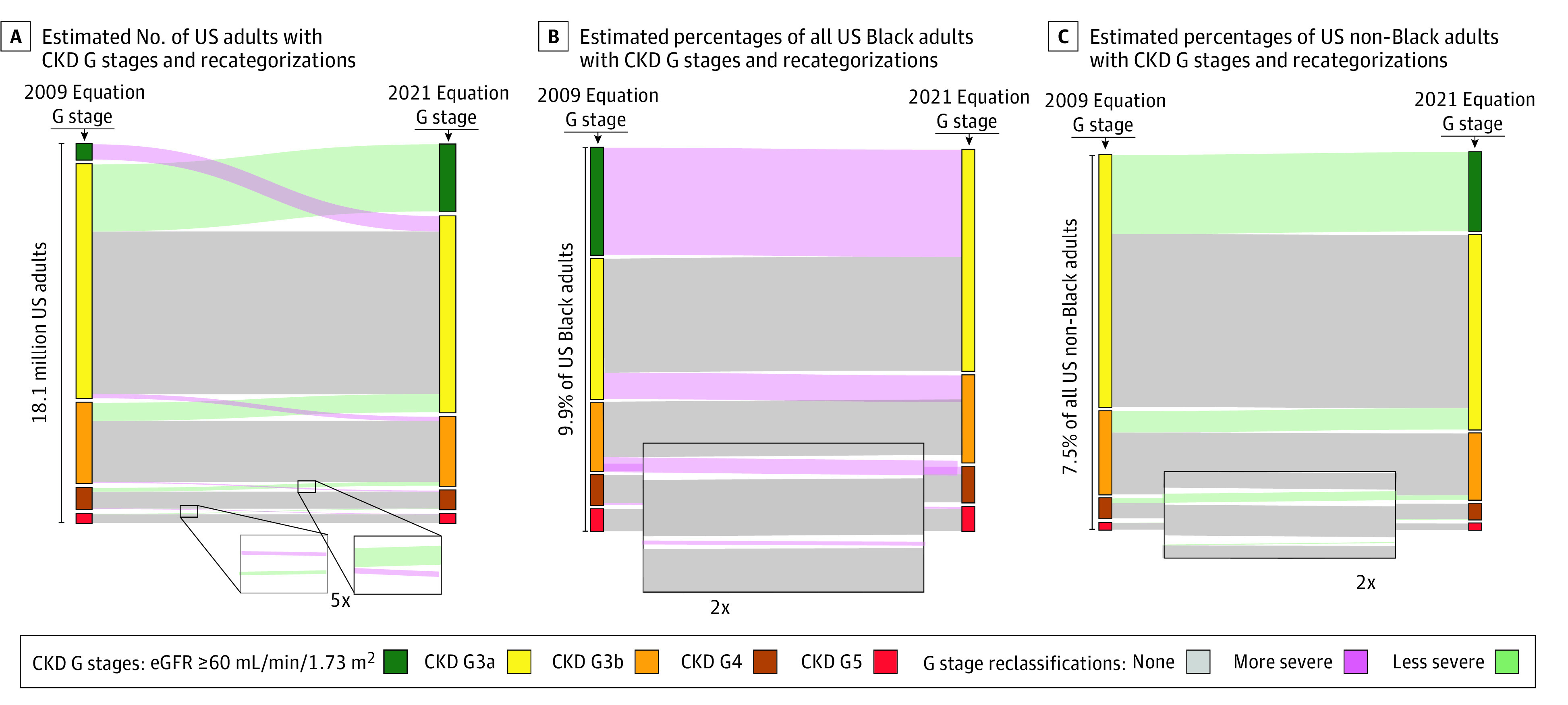
Estimated Glomerular Filtration Rate (eGFR) Category Change Estimates in the US Population From Chronic Kidney Disease (CKD)–Epidemiology Collaboration 2009 Equation to 2021 Equation The vertical widths of the bands represent proportions of individuals in (left axis) eGFR categories according to the 2009 equation, (right axis) eGFR categories according to the 2021 equation, and (middle) proportions belonging to connected left and right axis groups. Classifications use the Kidney Disease: Improving Global Outcomes CKD G classifications, based on eGFR, as follows: G1 or G2, 60 mL/min/1.73 m^2^ or greater; G3a, 45 to 59 mL/min/1.73 m^2^; G3b, 30 to 44 mL/min/1.73 m^2^; G4, 15 to 29 mL/min/1.73 m^2^; and G5, less than 15 mL/min/1.73 m^2^.

**Table.** Prevalence of Chronic Kidney Disease–Related Complications by Chronic Kidney Disease G-Stage Recategorization Group

G-stage recategorization group^a^	Proportion of individuals, % (95% CI)			
	Anemia	Hyperphosphatemia	Acidosis	Stage 2 hypertension
G3a to G2	6.2 (3.3-9.0)	6.9 (3.0-10.7)	9.2 (4.6-13.9)	28.5 (22.3-34.7)
G2 to G3a	6.6 (5.9-7.2)	3.6 (0.1-7.1)	14.8 (7.0-22.5)	43.3 (34.0-52.5)
Unchanged G3a	15.6 (12.3-18.9)	9.3 (6.6-11.9)	11.3 (8.3-14.3)	34.1 (29.4-38.8)
G3b to G3a	22.8 (13.1-32.6)	4.4 (0-8.9)	20.8 (8.0-33.7)	44.6 (32.1-57.1)
G3a to G3b	35.0 (21.3-48.8)	4.2 (0.0-10.7)	5.3 (0.0-11.2)	39.6 (22.5-56.7)
Unchanged G3b	34.4 (28.2-40.6)	13.8 (9.2-18.4)	15.3 (11.1-19.5)	41.3 (33.5-49.0)
G4 to G3b	34.1 (13.5-54.6)	11.8 (0-25.2)	15.3 (0.0-33.6)	49.9 (24.2-75.6)
G3b to G4	59.4 (25.8-93.1)	18.7 (0.0-41.3)	42.2 (5.1-79.3)	42.6 (5.8-79.3)
Unchanged G4	56.8 (46.5-67.1)	26.4 (17.0-35.8)	37.1 (26.1-48.1)	55.2 (44.1-66.2)
G5 to G4	82.6 (48.6-100)	34.1 (0.0-86.3)	66.6 (14.8-100)	82.6 (48.6-100)
G4 to G5	59.3 (0.9-100)	26.4 (0.0-79.9)	69.2 (17.8-100)	32.4 (0.0-86.3)
Unchanged G5	69.8 (54.4-85.3)	71.7 (57.2-86.3)	18.6 (7.3-23.9)	51.9 (34.7-69.2)

^a^ Indicates stage according to the Chronic Kidney Disease–Epidemiology Collaboration 2009 equation followed by recategorization according to the 2021 equation. Classifications use the Kidney Disease: Improving Global Outcomes Chronic Kidney disease G stage classifications, based on estimated glomerular filtration rate, as follows: G1 and G2, 60 mL/min/1.73 m^2^ or greater; G3a, 45 to 59 mL/min/1.73 m^2^; G3b, 30 to 44 mL/min/1.73 m^2^; G4, 15 to 29 mL/min/1.73 m^2^; and G5, less than 15 mL/min/1.73 m^2^.

## Discussion

Transitioning from the 2009 equation to the 2021 equation resulted in more than 5 million US adults changing CKD G stage classification, mostly in moderate CKD stages (between eGFR >60 mL/min/1.73 m^2^ and CKD stage G3a, ie, eGFR 45-59 mL/min/1.73 m^2^). The 2021 equation increased the prevalence of eGFR less than 60 mL/min/1.73 m^2^ among Black persons by 2.9 percentage points (7.1% to 9.9%), larger than the 2 percentage points reported using 1999 to 2002 NHANES data.^2^ The decrease in the prevalence of eGFR less than 60 mL/min/1.73 m^2^ among individuals identifying as non-Black of 1.6 percentage points (7.5% to 5.9%) was similar to that observed in 1999 to 2002 data.^2^ The prevalence estimates of CKD complications were qualitatively similar in reclassified and adjacent nonreclassified groups.

Limitations include use of single laboratory measurements. Limited numbers in some subgroups prevented precise estimates, including of complications, and prevented separate analysis of smaller racial and ethnic groups. Cystatin C levels are not available for these years.

In this study, estimation of eGFR using the 2021 CKD-EPI equation resulted in substantial reclassification in CKD G stage in US adults. However, CKD-related complication prevalence estimates were not substantially altered.

## Notes

Supplement. **eAppendix.** Supplementary Methods
eReferences.
