# Supplementary material for: Updated US Prevalence Estimates for Chronic Kidney Disease Stage and Complications Using the New Race-Free Equation to Estimate Glomerular Filtration Rate
Source: JAMA Netw Open. 2022 Feb 15;5(2):e220460. doi: 10.1001/jamanetworkopen.2022.0460 (PMC8848201; doi:10.1001/jamanetworkopen.2022.0460)
Supplement: Supplement. — eAppendix. Supplementary Methods eReferences. [file jamanetwopen-e220460-s001.pdf]

Supplemental Online Content

Walther CP, Winkelmayer WC, Navaneethan SD. Updated US prevalence estimates for chronic kidney disease stage and complications using the new race-free equation to estimate glomerular filtration rate. *JAMA Netw Open.* 2022;5(2):e220460.
doi:10.1001/jamanetworkopen.2022.0460

eAppendix. Supplementary Methods

eReferences.

This supplemental material has been provided by the authors to give readers additional information about their work.

© 2022 Walther CP et al. *JAMA Network Open.*

eAppendix. Supplementary Methods

Study Population

Self-reported multi-racial status is not identifiable from the public NHANES datasets.[1]

Analysis

We estimated weighted totals and proportions for those who met inclusion criteria and for subgroups in our analysis. To enable more stable estimates, we combined four 2 year data cycles (8 years), 2011-2018, and adjusted the weights accordingly, enabling estimates of totals and proportions of the US population with appropriate variances.[2] Inclusion in the study required participation in the NHANES exam phase, and thus we used the examination weights.[3] We used survey methods for all analyses, to account for clustering, stratification, and weighting. Taylor series linearization was used for estimation of variances.[3,4]

To try to reduce possible bias, multiple imputation was used, under the Missing At Random (MAR) assumption. Twenty imputations were performed using chained equations and predictive mean matching, using analytic variables (serum creatinine, hemoglobin, serum bicarbonate, serum phosphate, systolic blood pressure, diastolic blood pressure, age, sex, and Black *versus* non-Black race) and design variables (weights, composite stratum x cluster indicator variable).[4]. Estimated glomerular filtration rates were passively imputed using the imputed serum creatinine levels. All estimations used the multiple imputations according to the combination rules of Rubin.[5]

NHANES data sets reports ages ≥ 80 years as 80 years to ensure anonymity. The average age of those reported as 80 years is 85 years for 2015-16,[1] which we used as the approximation for people with age recorded as 80 or older for purposes of eGFR estimation. All analyses were performed using Stata 14.2 (www.stata.com). The Sankey diagram was created using the R version 4.0.2 (www.r-project.com) and the package networkD3.[6]

© 2022 Walther CP et al. *JAMA Network Open.*

eReferences

1. National Center for Health Statistics. National Health and Nutrition Examination Survey Publications and Products. 2020; https://www.cdc.gov/nchs/nhanes/nhanes_products.htm. Accessed November 23, 2020.
2. Centers for Disease Control and Prevention. National Health and Nutrition Examination Survey: analytic guidelines, 2011–2014 and 2015–2016. *National Center for Health Statistics, editor Atlanta, GA: Centers for Disease Control and Prevention.* 2018.
3. Chen T-C, Clark J, Riddles MK, Mohadjer LK, Fakhouri TH. National Health and Nutrition Examination Survey, 2015− 2018: sample design and estimation procedures. 2020.
4. Heeringa SG, West BT, Berglund PA. *Applied Survey Data Analysis.* 2nd ed. Boca Raton, FL: CRC Press; 2017.
5. Rubin DB. *Multiple imputation for nonresponse in surveys.* Vol 81: John Wiley & Sons; 2004.
6. *networkD3: D3 JavaScript Network Graphs from R* [R package]. Version 0.4 2017.
